# Remote Welfare Monitoring of Rodents Using Thermal Imaging

**DOI:** 10.3390/s18113653

**Published:** 2018-10-28

**Authors:** Carina Barbosa Pereira, Janosch Kunczik, Leonie Zieglowski, René Tolba, Ahmed Abdelrahman, Dietmar Zechner, Brigitte Vollmar, Heike Janssen, Thomas Thum, Michael Czaplik

**Affiliations:** 1 Department of Anesthesiology, University Hospital RWTH Aachen, Pauwelsstrasse 30, 52074 Aachen, Germany; jkunczik@ukaachen.de (J.K.); mczaplik@ukaachen.de (M.C.); 2 Institute for Laboratory Animal Science and Experimental Surgery, University Hospital RWTH Aachen, Pauwelsstrasse 30, 52074 Aachen, Germany; lzieglowski@ukaachen.de (L.Z.); rtolba@ukaachen.de (R.T.); 3 Institute for Experimental Surgery, Rostock University Medical Center, Schillingallee 69a, 18057 Rostock, Germany; Ahmed.Abdelrahman@med.uni-rostock.de (A.A.); dietmar.zechner@uni-rostock.de (D.Z.); brigitte.vollmar@uni-rostock.de (B.V.); 4 Institute of Molecular and Translational Therapeutic Strategies (IMTTS), Hannover Medical School, Carl-Neuberg-Str. 1, 30625 Hannover, Germany; Janssen.Heike@mh-hannover.de (H.J.); thum.thomas@mh-hannover.de (T.T.); 5 Excellence Cluster REBIRTH, Hannover Medical School, Carl-Neuberg-Str. 1, 30625 Hannover, Germany

**Keywords:** animal research, remote monitoring, vital signs, respiratory rate, locomotor activity, motion heat maps, thermal imaging, infrared thermography

## Abstract

Animal research has always played a crucial role in various medical and scientific breakthroughs. They offer, inter alia, insights into diseases mechanisms, genetic predisposition to a disease, and drug therapy. However, the use of animals for medical research is a cause of major controversies and debates in modern science. To warrant high bioethical standards, new directives have been being adopted to replace animal research whenever possible, to reduce the number of animals, and to refine the procedures to minimize stress and pain. Here, we present two new approaches, based on thermal imaging (a remote and passive technology), to assess respiratory rate (RR) as well as exploratory behavior and general activity in rodents. In animal research, these parameters are gold standards for welfare assessment. The approaches were validated in a study conducted with both rats and mice. To test the feasibility of our algorithm to estimate RR, thermal videos from anesthetized rodents were acquired. The capability of the second approach to monitor activity was tested with videos of Open Field tests. Regarding RR, a high agreement between thermal imaging and gold standard (electrocardiography-derived RR) was achieved. The mean relative error averaged 0.50 ± 0.15 breaths/min and 4.55 ± 2.94 breaths/min for rats and mice, respectively. The second approach was capable of monitoring and tracking the activity of the rodents very well. This paper demonstrates that thermal imaging is a promising and relevant alternative for monitoring of RR and activity in rodents, thus contributing to the remote assessment of animal welfare.

## 1. Introduction

“*Animal research and testing has played a part in almost every medical breakthrough of the last century. It has saved hundreds of millions of lives worldwide and is vital to our National Health Service.*” Home Office Minister Joan Ryan.

Animal research has led to numerous medical and scientific breakthroughs [1]. Animal studies have been offering insights into several genetic predispositions to diseases, their mechanisms, pathogenic agents and their interaction with the human body, drug therapy, among others [1,2]. In fact, they had a significant contribution in the major achievements of modern medicine, including most vaccines, treatment of type I diabetes by insulin, magnetic resonance imaging, novel cancer treatments, minimally invasive surgical techniques, etc. [3]. Animal models, foremost using rodents, are commonly used in biomedical research because they replicate diverse human physiological and pathophysiological processes very well [4]. The medical and scientific advances were not only meaningful for human medicine but also for veterinary medicine, since dozens of diseases, e.g., cancer and epilepsy, affect both animals and humans. Therefore, treatments (vaccines, antibiotics, etc.) applied to humans can also benefit animals [5].

However, using animal models for scientific and medical advances is ethically controversial [1,6]. To warrant high bioethical standards, new directives have been being adopted on the protection of animals used for research. For example, in 2010 the European Parliament adopted a Directive 2010/63/EU to ensure animal welfare; it is based on three main principles—the **3R-Principles**: **replacement**, **reduction** and **refinement** [1,7,8,9]. The aim is to **replace** animal testing with alternatives or complementary methods, **reduce** the number of animals used in research experiments, and **refine** the procedures to minimize stress and pain, and consecutively, improve the welfare of the animals under study [9,10].

To assess the animals’ well-being, a continuous monitoring of their vital parameters (heart rate; HR and respiratory rate; RR), emotional state and locomotion capability is fundamental [11]. In chronic animal experiments, vital signs can be assessed by implanting a telemetry sensor. However, this initial surgical implantation procedure might lead to discomfort, stress, pain, chronically limited mobility, and as a result compromise animal welfare. In acute experiments with animals undergoing anesthesia, HR and RR are monitored using electrocardiography (ECG) or photoplethysmography (PPG). Here, the required wires are often hindering for the researcher, working with the animal. Other parameters including emotional state, locomotion capability or pain are currently assessed subjectively [12,13,14].

In recent years, there have been increasing demands for unobtrusive and contactless monitoring modalities to **refine** animal experiments [15,16]. Thermal imaging, also infrared thermography (IRT), emerged as a promising monitoring technology in a wide spectrum of medical fields, including assessment of HR [13,17] and RR [18,19], monitoring of thermal regulation [20], as well as observation of circulation and perfusion dynamics [21]. Thermal imaging is a remote and passive monitoring technique that detects and records the radiation naturally emitted from a body. Hence, IRT neither uses potentially harmful radiation nor needs a light source [21]. Commonly, thermal cameras operate on the short-wave (e.g., 2.5–3 μm [22]), mid-wave (e.g., 3–5 μm [18]) or on the long-wave infrared spectrum (e.g., 7–14 μm [21]).

The current paper presents methods to assess rodents’ welfare during research experiments using thermal imaging. The first approach allows to monitor RR, based on thorax movements due to respiratory activity. Respiration is necessary for assessing the physiological and psychological state [15]. According to Cretikos et al., an abnormal RR is one of the earliest indicators of physiological distress [23]. The second approach is capable of tracking the rodents and compute position, velocity as well as motion heat maps during Open Field tests (OFTs). The movement and location of the rodents during the gold standard OFTs is important to assess both exploratory behavior and general activity, which are major markers for anxiety, stress and pain [24,25].

## 2. Methods

### 2.1. Estimation of Respiratory Rate

**Figure 1:**
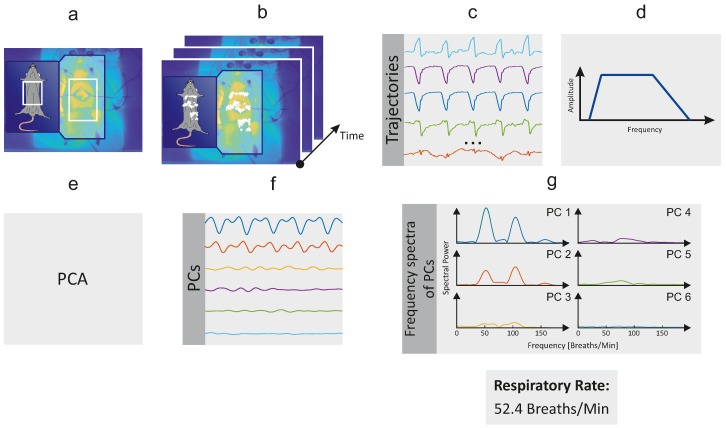
Schematic overview of the RR algorithm. (**a**) Selection of region of interest (ROI); (**b**) Detection and tracking of feature points; (**c**) Extraction of feature points’ trajectories; (**d**) Temporal filtering; (**e**) Blind source separation via principal component analysis (PCA); (**f**) Rank principal components (PCs) based on their variance; (**g**) Computation of frequency spectra and estimation of RR.

In this study, the rodents’ RR was estimated using a motion-based computer vision method. This approach relies on the fact that mechanical chest movements accompany the respiratory cycle. The algorithm can be divided in six major steps, which are described in detail below. Figure 1 provides a schematic overview of the algorithm. It was programed in MATLAB (MATLAB 2018a, The MathWorks Inc., Natick, MA, USA) and the data were analyzed offline.

#### 2.1.1. Selection of Region of Interest

In the first step, the region of interest (ROI), containing the thorax, was manually selected in the first frame of the video.

#### 2.1.2. Image Preprocessing

The animal was segmented from the background using a multilevel Otsu’s algorithm that computes optimal threshold values using discriminant analysis [26]. In addition, the contrast of the thermograms was improved by linearly stretching the initial gray levels to a new range. Feature points were identified within the ROI in the first frame of the video and tracked over time. Both methods were performed for each frame of the thermal video.

#### 2.1.3. Motion Tracking

To track motion, *N* = 100 distinctive feature points (such as corners and textured areas) were identified within the ROI and tracked over time. In this paper, the approach of Shi and Tomasi [27], well-known as the Shi-Tomasi corner detector, was used. Please note that this step was only performed in the first frame of the video sequence. The positions of the *N* feature points were given as input to the Lucas-Kanade method. It tracked their trajectories over time using optical flow. To isolate only relevant motions, the signal quality of the trajectories was taken into consideration. Feature points presenting a poor texture can lead to an inaccurate tracking and, consecutively, generate erratic trajectories. To preserve only the most stable feature points, those points whose trajectories between consecutive frames exceeded a predefined percentile (25%) were discarded. Afterwards, motion trajectories were divided into their vertical and horizontal components. The component containing the main chest movement was selected for further analysis.

#### 2.1.4. Temporal Filtering

Not all trajectories related to the movement of the chest are caused by respiration. Thus, the signal frequencies were constrained to the expected ranges of RR to remove noise. Under normal conditions, the RR of mice ranges from 91–216 breaths/min and that of rats ranges from 71–146 breaths/min [28]. Since anesthesia decreases RR, band-pass FIR (finite impulse response) filters with a passband band of [1 4.6] Hz and [0.6 3.3] Hz were applied, respectively. They were designed using the Parks-McClellan algorithm. The upper cutoff frequency was set to include the RR harmonics, as they offer relevant information for a correct peak detection.

#### 2.1.5. Principal Component Analysis Decomposition

The chest movement due to respiration is the underlying signal of interest. However, there are other sources which were not filtered in the previous step and may affect the trajectories of the feature points. Blind source separation was applied via principal component analysis (PCA) in order to isolate the set of main dimensions along which the position of the chest varies. In short, this approach decomposes the initial dataset into a new set of linearly uncorrelated variables. For a set of *N* feature point trajectories, PCA finds principal components. These are ordered so that the first few include most of the variation contained in the original dataset [29]. Empirical evidence demonstrated that, in this case, only the first six components are necessary for further analysis.

#### 2.1.6. Principal Component Selection

In the last step, the component containing the respiratory signal is extracted based on signal periodicity. Signal periodicity is analyzed using the frequency spectra. However, before applying the fast Fourier transform (FFT), the six principal components were hamming windowed, to reduce edge effects, and zero padded. The periodicity of the components was quantified by applying the peak-to-total ratio approach: the ratio of the frequencies within a range of 0.05 Hz around the dominant frequency and a range of 0.05 Hz around its first harmonic in the frequency spectrum to the total spectral density. Whereas a high periodicity denotes a signal with a dominant frequency, a low periodicity stands for a signal containing aperiodic noise. Directly after selecting the principal component with the highest periodicity, the clearest main frequency was chosen as RR. Lastly, to avoid outliers in the RR signal, a median value of the last five seconds was computed. Regardless of a slight delay in the signal, a more accurate estimation of this vital parameter can be achieved.

### 2.2. Assessment of Exploratory Behavior and General Activity

Exploratory behavior and general activity are common parameters for assessment and evaluation of, inter alia, fear-related behavior, and sickness. To illustrate spatial distributions of exploratory behavior, motion heat maps are commonly used in the scientific community. In this paper, a new approach to assess both exploratory behavior and general activity of rodents using thermal imaging is presented. The algorithm can be divided in three major steps, which are described in detail below. It was programed in MATLAB (MATLAB 2018a, The MathWorks Inc., Natick, MA, USA) and the data were analyzed offline.

#### 2.2.1. Selection of Region of Interest

The first step involves defining the ROI, i.e., the rodent, in the first frame of the thermal video. In thermal imaging, this task is indeed easier as compared to conventional camera sequences, since the rodent is the warmest region in the thermogram.

#### 2.2.2. Motion Tracking

To track the rodent (ROI), the approach developed by Mei and Ling [30], was used. It integrates sparse representation into a particle filter-based object tracker. Two main models were included in the tracker: a state transition model and an observation model. The former uses conditional density to estimate the correlation of a state transition between consecutive frames and selects the candidate samples in the current video frame. The latter compares the similarity between target candidate and target model, i.e., it works as a target/background classifier. To detect the ROI in the current frame, each single target candidate was sparsely represented in the target template space and sparsity (or sparse representation) could be found using *ℓ*1 minimization. The candidate with the smallest projecting error was considered the tracking result, i.e., the position of the ROI in the current frame.

#### 2.2.3. Motion Parameters and Heat Maps

During the tracking, the positon and velocity of the rodent was assessed continuously. In addition, a heat map plot, illustrating cumulative time spent in different parts of the experimental arena, were computed.

## 3. Experimental Protocol

The current work was performed in cooperation with three further research groups as a part of the research unit project (composed of several research groups all over Germany) entitled “Severity Assessment in Animal Based Research”. Therefore, the data presented in this paper have three different sources.

### 3.1. Collection of Thermal Videos for Assessment of Respiratory Rate

To investigate the performance of our approach to assess RR in rodents, two studies, one in mice (at the Institute of Molecular and Translational Therapeutic Strategies, Hannover Medical School) and the other in rats (at the Institute for Laboratory Animal Science and Experimental Surgery, University Hospital, RWTH Aachen University), were carried out. During the collection of thermal videos, the rodents were under isoflurane anesthesia. Mice were anesthetized with 3 vol % isoflurane and 0.8 L/min of oxygen by inhalation. Afterwards, anesthesia was maintained by decreasing the dose to 0.8–2 vol %. Rats, in turn, were anesthetized with 5 vol % isoflurane and 5 L/min of oxygen by inhalation. Anesthesia was maintained by reducing the dose of anesthetic to 2 vol % and 2 L/min of oxygen.

**Figure 2:**
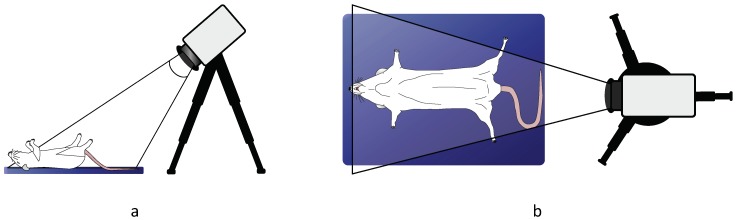
Illustration of the measurement setup for RR assessment: (**a**) side view and (**b**) top view.

Thermal videos of 2-min duration each were recorded using a long-wave infrared camera, VarioCAM^®^ HD head 820S/30 mm (InfraTec GmbH, Dresden, Germany). This detects wavelengths in the spectral range of 7.5–14 μm and presents a thermal sensitivity better than 0.05 K at 30 °C. Thermograms were acquired with a frame rate of 60 fps and a spatial resolution of 640 × 480 pixels. The thermal camera was set atop a tripod, which was strategically placed to provide a frontal view of the rodent as illustrated in Figure 2. ECG was measured simultaneously as gold standard. In the study involving mice, the system Vevo 2100 Imaging System (VisualSonics, Toronto, ON, Canada) was used. Since this did not provide an output of vital signs waveforms, the RR values (displayed on the device) were manually annotated. In rats, ECG was assessed using the data recording system PowerLab and the LabChart data analysis software (ADInstruments, Dunedin, New Zealand). RR was computed from the ECG baseline wander, which is mainly influenced by thoracic impedance changes due to respiration.

The studies were approved by the governmental institution “*Niedersaechsisches Landesamt für Verbraucherschutz und Lebensmittelsicherheit*” (Germany; 33.12-42502-04-15/1978) and “Landesamt für Natur, Umwelt und Verbraucherschutz NRW” (Germany; 84-02.04.2017.A304). They were performed according to the declaration of Helsinki and the guiding principles in the care and use of animals.

### 3.2. Collection of Thermal Videos during Open Field Tests

**Figure 3:**
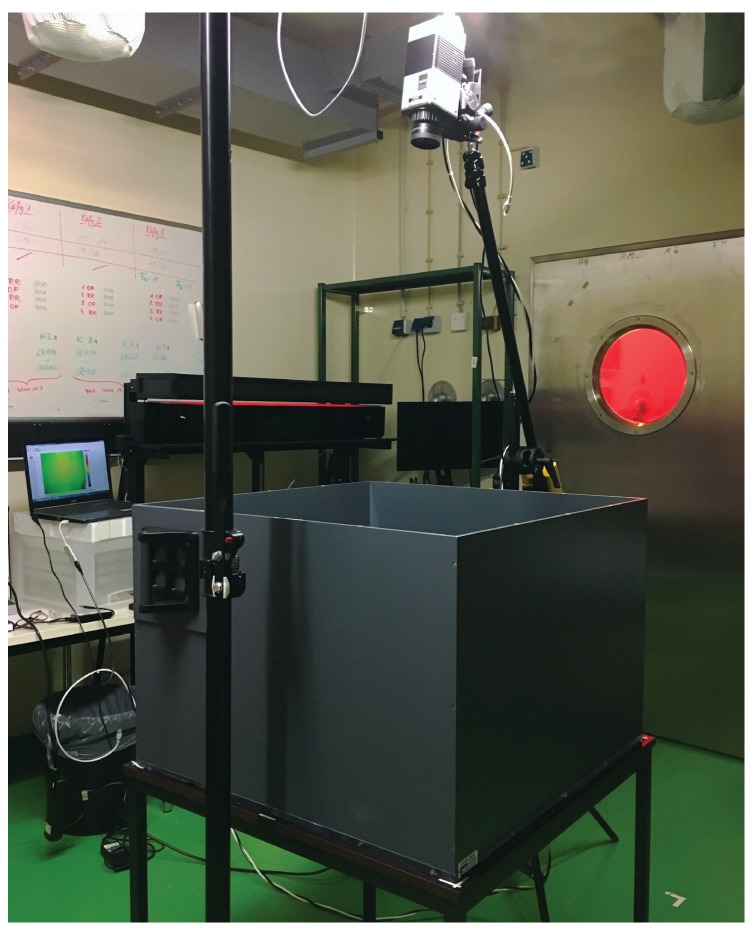
Photo of the setup used for the OFTs in rats.

To investigate the capability of our algorithm to provide accurate color heat maps of activity, OFTs with five mice and five rats were carried out. While the study in mice was performed at the Institute for Experimental Surgery, Rostock University Medical Center, the data from rats was collected at the Institute for Laboratory Animal Science and Experimental Surgery, University Hospital, RWTH Aachen University. In the OFTs, mice and rats were individually placed in a square/rectangular arena surrounded by high walls to prevent escape. The animals could freely move freely in the open field before being returned to their home cage. In the experiment involving mice, 30-s thermal videos were acquired. The arena had a size of 167 × 225 mm. In the experiment involving rats, 5-min videos were analyzed. Here, a larger open field cage, 720 × 720 mm, was used. The thermal videos were recorded using the VarioCAM^®^ HD head 820S/30 mm (InfraTec GmbH, Dresden, Germany) as well. It was mounted on a tripod crane, which was strategically placed so that the open field arena fits the camera’s view. Figure 3 shows a photo of the experimental setup.

The previous studies were approved by the governmental institution “*Landesamt für Landwirtschaft, Lebensmittelsicherheit und Fischerei Mecklenburg-Vorpommern*” (Germany; 7221.3-1-002/17-5 and 7221.3-2-039/14-15) and “*Landesamt für Natur, Umwelt und Verbraucherschutz NRW*” (Germany; 84-02.04.2017.A304), respectively. They were performed according to the declaration of Helsinki and the guiding principles in the care and use of animals.

## 4. Results

### 4.1. Estimation of Respiratory Rate

Table 1 shows the performance of the approach developed for estimation of RR in videos of anesthetized rats. On average, the RR of the rodents hovered around 51.85 ± 6.56 breaths/min. Comparison between two monitoring techniques (thermal imaging and gold standard) demonstrated a root-mean-square error (RMSE) of 0.35 ± 0.09 breaths/min. In addition, the mean relative RR error, $\bar{\varepsilon }$, averaged 0.50 ± 0.15% and the spread of the relative error (computed using the 90th percentile of the relative errors, $\varepsilon_{90}$) was 1.05 ± 0.32%. High correlations between RR estimated with thermal imaging and RR derived from ECG were observed; the mean correlation averaged 0.97 ± 0.01 (all *p*-values were smaller than 0.05).

**Figure 4:**
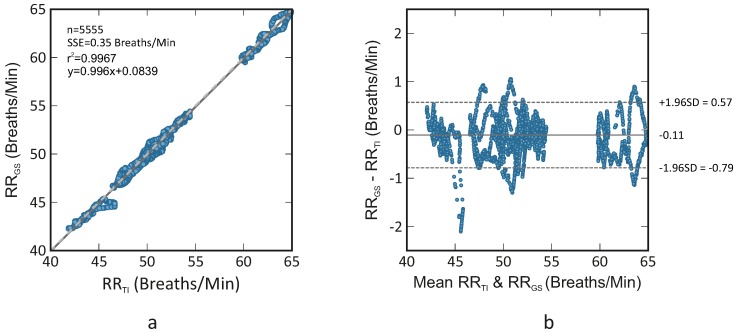
Correlation plot (**a**) and Bland-Altman plot (**b**) comparing RR assessed using thermal imaging (RR${}_{TI}$) and RR assessed using ECG (RR${}_{GS}$); the plots comprise the data of all five rats. The former shows a R-squared of 0.967 and a sum of squared errors of 0.35 breaths/min. The latter demonstrates a bias of −0.11 breaths/min (solid line) and the 95 % limits of agreement vary between −0.79 breaths/min and 0.57 breaths/min (dashed lines).

Figure 4 displays a correlation plot and a Bland-Altman plot comparing both monitoring techniques (thermal imaging and gold standard); they comprise the data from all five rats. According to the results, the R-squared (coefficient of determination) was 0.967 and the sum of squared errors (SSE) averaged 0.35 breaths/min. The Bland-Altman plot showed a mean difference of −0.11 breaths/min. Its limits of agreement ranged from −0.79 breaths/min to 0.57 breaths/min.

**Figure 5:**
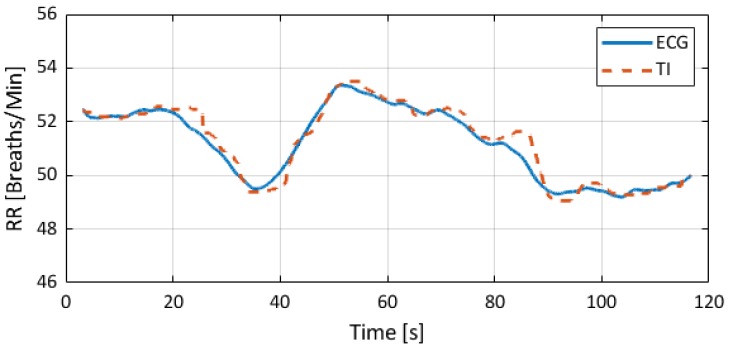
Estimated RRs. The solid line stands for the gold standard (RR derived from ECG), and the dashed line corresponds to the RR obtained with thermal imaging.

Figure 5 presents an example of RR estimated with thermal imaging (dashed line) as well as the RR corresponding to the gold standard (solid line); these representative signals are from the animal R1. Video S1 (Supplementary Material) provides a short animation showing the performance of the algorithm. Also here, the data from animal R1 were used.

Table 2 presents the performance of the algorithm developed for estimation of RR in videos of anesthetized mice. On average, the RR of the rodents stayed around 113.40 ± 29.42 breaths/min. The mean relative RR error ($\bar{\varepsilon }$) amounted 4.55 ± 2.94%. Video S2 (Supplementary Material) provides a short animation of RR estimation in thermal videos of mice. In this illustrative example, the data from animal M1 were used.

### 4.2. Assessment of Exploratory Behavior and General Activity

**Figure 6:**
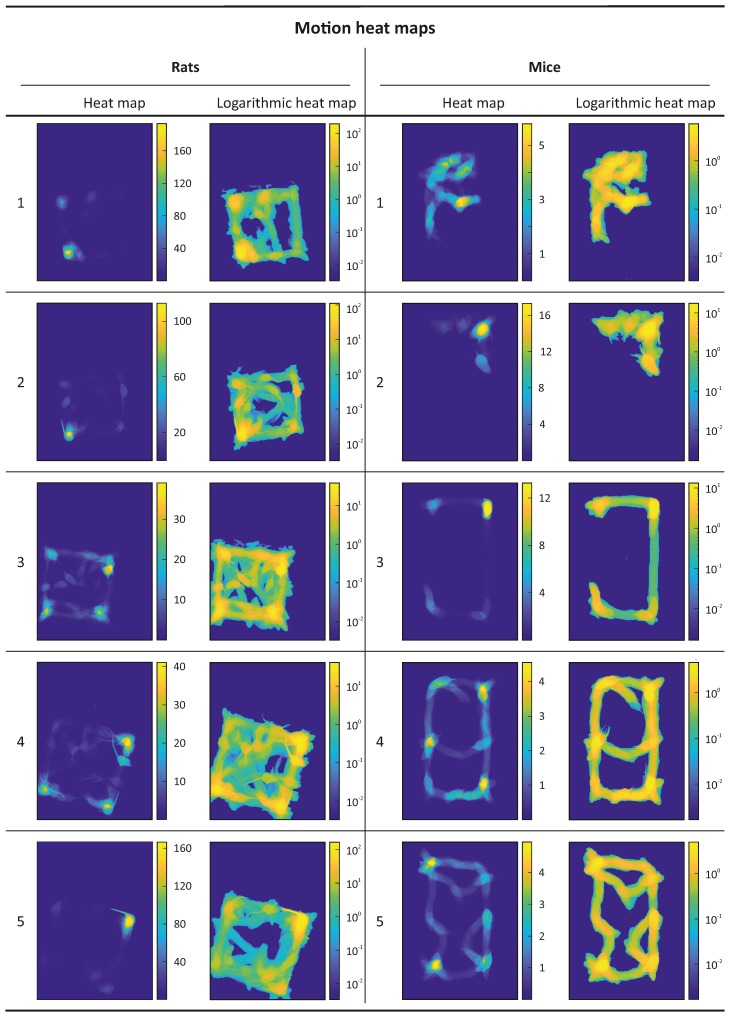
Motion heat maps and logarithmic motion heat maps representing the cumulative time that the rodents spent in the different parts of the arena. Yellow denotes more time and blue less or no time. The time periods are given in seconds.

The results from the assessment of exploratory behavior and general activity are shown in the form of motion heat maps in Figure 6. Their color scheme indicates the cumulative time spent in different parts of the arena; yellow denotes more time and blue less or no time. For larger periods of time, the complete path traveled by the animal is often not visible, therefore logarithmic heat maps are also represented in Figure 6. Videos S3 and S4 (as Supplementary Material) are illustrative examples of the performance of the proposed approach in rats and mice, respectively. They correspond to the rat 1 and mouse 3, respectively.

## 5. Discussion

The current paper aims to evaluate the capability of thermal imaging to monitor RR and locomotor activity, which are important parameters for evaluation of rodents’ welfare. To test the performance of both approaches, studies in mice and rats were performed.

RR is not only an important parameter for assessment of health condition and physiological deterioration in humans, but also in animals. Breathing disorders, identified by an abnormal rate, respiratory sound or atypical waveforms (abnormal depth and/or rhythm), are early and strong markers of serious complications. They can be, additionally, associated with fear, anxiety, panic or even pain. Therefore, in line with HR, a continuous monitoring of RR is highly requested in animal research, especially using contactless and passive techniques such as thermal imaging. In this paper, we presented an approach, which extracts RR from the chest movement.

Table 1 and Table 2 showed a good agreement between gold standard (ECG-derived RR) and thermal imaging for both species. In rats, the RMSE and correlation averaged 0.35 ± 0.09 breaths/min and 0.97, respectively. The correlation plot and Bland-Altman plot of Figure 4 corroborate the good agreement between both monitoring technologies, with the limits of agreement ranging from −0.79 breaths/min to 0.57 breaths/min. In this group, the mean RR varied between 40 breaths/min and 65 breaths/min, approximately. No difference in the standard deviation of errors was observed for the covered range. Table 2 showed the mean relative errors for RR estimation in mice; they varied around 4.55 ± 2.94%. By comparison with rats, higher relative errors were obtained (rats: 0.50 ± 0.15%, mice: 4.55 ± 2.94%). Unfortunately, in the study involving mice, the measurement data system had no analog output, thus just the mean RR was compared. In fact, this might be a plausible reason for the higher errors. Nevertheless, also in this case, a good agreement between both techniques was obtained. In general, Table 1 and Table 2 report relative lower RRs. This was a consequence of the anesthesia, which decreases per se both RR and tidal volume.

In 2018, Mutlu et al. [15] presented an approach for respiration monitoring in head-restrained rodents using thermal imaging. In contrast to our work, it is based on the fact that temperature around the nose varies during the respiratory cycle. While cold air from the environment is inhaled during inspiration, warm air from the lungs is exhaled during expiration. To validate the algorithm, a study in six mice was carried out. Instead of ECG, intranasal pressure was used as gold standard. In general, comparable results were obtained. The median error rate averaged 2.8 ± 0.4%. Albeit this is a good alternative to monitor respiratory activity, there is a drawback regarding the measurement principle that should be addressed. In the algorithm proposed by Mutlu et al. [15], the nostrils of the rodent must be always in the field of view of the camera. By contrast, in our approach the position of the camera can be flexible. The algorithm works well with both frontal or side views of the subject. The only main requirement is that the thorax or belly must be partly visible.

Also in 2018, Vainer [11] proposed an approach, denominated “sorption-enhanced infrared thermography” (SEIRT), for robust assessment of respiration in mammals. According to the author, it combines the advantages of IRT and chemical physics in a single method. In [11], two novel techniques were used to quantify respiratory activity; one extracted RR from the chest movement and the other from SEIRT. Both techniques were based on IRT. The advantages of SEIRT were reinforced by comparing it with a popular approach based on the temperature modulation around the open nostrils. When SEIRT is used, a breathing sorption indicator, e.g., a hydrophilic material (such as cotton), is required to be placed close to the nostrils or/and mouth. To increase the magnitude of the ordinary IRT-based signal associated with thorax movement, the author used a movement amplitude enhancer, i.e., an elastic cantilever, which was leant against the thorax. In both approaches, the breathing intervals were computed by finding the peaks in the extracted signal. For validation purposes, data from 42 subjects, 49 rats and 4 minipigs were acquired. The results for rats demonstrated a great correlation between the breathing intervals of both approaches with a correlation of 0.98. However, unfortunately, no gold standard was used in the study. If on the one hand, the two techniques are very promising since they are highly sensitive, simple, flexible (independent from the camera’s position) and robust, on the other hand, the SEIRT is dependent on an external component (breathing sorption indicator), and the thorax movement-based approach preferably requires an elastic cantilever.

In the vast majority of current chronic animal studies, vital signs are not monitored continuously. As mentioned previously, telemetry systems can be used to collect these data, but they require an initial surgical intervention for implantation, which causes additional stress and pain to the animals. In acute animal studies, vital signs are monitored using standard measurement techniques, such as ECG, photoplethysmography, etc. These techniques are feasible, but they require the attachment of sensors and cables to the subject’s body. Therefore, one of the major goals in modern animal research is to **refine** (3Rs Principle) the procedures to minimize stress and pain as far as possible. We believe that thermal imaging can contribute to the **refinement** of animal trials. Its remarkable features and capabilities make this technology a promising alternative to the currently available measuring modalities. Despite of the promising results, the current method presents a major drawback, the ROI (thorax) must be selected manually at the beginning of the analysis. Thus, a next version of the algorithm should be capable of detecting the thorax automatically; this can be achieved using a machine learning approach for visual object detection.

In addition, we presented an algorithm capable of monitoring activity and exploratory behavior in rodents. It allows to track the positon and velocity of the animal. For visualization purposes, heat maps can also be estimated. Motion heat maps are meaningful during the examination of behavioral patterns, because parameters such as (1) total distance travelled, (2) time spent in the central and peripheral zone, (3) time of immobility, (4) distance travelled in the central and peripheral zones, among others, are indicators of the physiological and psycho-sociological status of experimental subjects. For instance, low ambulation indicates commonly fear (or anxiety). In this work, the performance of the motion tracking algorithm was assessed visually. Video S3 and Video S4 are two examples demonstrating the great outcome of our approach in tracking the animal and in estimating the motion heat maps.

Currently, there are commercially available systems, composed of a CMOS (Complementary Metal-Oxide Semiconductor) or a CCD (Couple-Charged Device) camera, capable of tracking the animal motion during OFTs. Despite of the good performance, they present major drawbacks, which can be compensated with thermal imaging. First, thermal imaging does not depend on any light source. In contrast, near-infrared and visible imaging systems require a light source to produce an image. This property is especially important for the monitoring of the circadian rhythms. Second, the fear or anxiety response of an animal exposed to a potentially dangerous environment is accompanied by high defecation and urination. These physiological responses are visible in thermal imaging in the form of hot spots, and could be evaluated as well. Please note that these responses can be differentiated from the animal itself. Lastly, animals can be much better segmented from the significantly colder background when using IRT. In contrast to visual video solutions, the color of the selected strain, the monitoring cage, litter and fodder does not play a role.

IRT is still an expensive technique, especially when compared to visible imaging systems. However, it holds an important advantage over other technologies: it is independent from light. A major challenge when using “regular” cameras in a real setting is to remove aliased components from artificial light, such as fluorescent lights. The 100 Hz flicker frequency component (100 Hz in Europe and 120 Hz in the US) can be aliased down to frequencies similar to the heart beat. This occurs because the image is sampled at the camera’s frame rate (mostly, 30 Hz), which is much lower than the the above-mentioned flicker frequencies. Moreover, we may not forget that in recent years there was a significant increase in the amount of IRT applications, mainly in consumer-oriented applications, like driver vision enhancement and home security. This phenomenon led to an increase in production volumes with consequent decrease in prices. As other technologies (e.g., radar), we believe that IRT cameras will become affordable and even better.

## 6. Conclusions

In this joint research project, we demonstrated the capabilities of camera-based data assessment for the sake of animal health and, especially, potential refinements. In our opinion, this is a fine step forward in terms of a more objective severity assessment during animal trials.

The current paper focused on motion profiles, activity and RR. In general, it was feasible to obtain the designated data with an excellent accuracy as compared to reference. The combination of these parameters, exploratory activity and vital signs, may contribute not only to objectively detect stress or pain but also to quantify their levels.

Further features/parameters must be integrated including measuring pulse rate, perfusion, tissue inflammation. The overall aim should be to take maximum advantage of IRT by using it as a multi-function monitoring device/tool in animal research.

Another very important point that should be tested in the future is the capability of the algorithm to measure RR in moving animals. Here, the tracking algorithm used in Section 2.2 must be combined with the RR approach. Finally, apart from the application of severity assessment in rodents, there is also a huge potential for further laboratory animals—especially when being used in chronic animal models lasting for several days, weeks or months.

## Figures and Tables

**Table 1:** Results for RR estimation in thermal videos from rats.

Rodent ID	Mean RR (Breaths/Min)		RMSE	$\bar{\varepsilon }$ (%)	$\varepsilon_{90}$ (%)	Correlation
	GS	TI	(Breaths/Min)			
R1	51.17	51.27	0.32	0.44	0.92	0.98
R2	53.58	53.58	0.21	0.28	0.66	0.96
R3	43.57	43.70	0.38	0.52	1.05	0.95
R4	49.38	49.53	0.42	0.67	1.54	0.98
R5	61.54	61.69	0.43	0.57	1.08	0.97
**Mean ± SD**	**51.85 ± 6.56**	**51.95 ± 6.56**	**0.35 ± 0.09**	**0.50 ± 0.15**	**1.05 ± 0.32**	**0.97 ± 0.01**

GS—gold standard, TI—thermal image, $\bar{\varepsilon }$—mean relative error, $\varepsilon_{90}$—90th percentile of the relative errors.

**Table 2:** Results for RR estimation in thermal videos from mice.

Rodent ID	Mean RR (Breaths/Min)		$\bar{\varepsilon }$ (%)
	GS	TI	
M1	130.00	131.46	1.12
M2	132.00	144.13	9.19
M3	70.00	67.41	3.70
M4	96.00	99.69	3.84
M5	139.00	145.81	4.90
**Mean ± SD**	**113.40 ± 29.42**	**117.70 ± 33.66**	**4.55 ± 2.94**

GS—gold standard, TI—thermal image, $\bar{\varepsilon }$—mean relative error.

## Supplementary Materials

The following are available online at http://www.mdpi.com/1424-8220/18/11/3653/s1, Video S1: Signal processing for assessing respiratory rate in rats. Video S2: Signal processing for assessing respiratory rate in mice. Video S3: Computation of motion heat maps using thermal videos of rats. Video S4: Computation of motion heat maps using thermal videos of mice.

## Abbreviations

The following abbreviations are used in this manuscript:

$\bar{\varepsilon }$	Mean relative error
$\varepsilon_{90}$	90th percentile of the relative errors
CCD	Couple-Charged Device
CMOS	Complementary Metal-Oxide Semiconductor
ECG	Electrocardiography
FFT	Fast Fourier transform
FIR	Finite impulse response
GS	Gold standard
HR	Heart rate
IRT	Infrared thermography
OFT	Open Field test
PC	Principal component
PCA	Principal component analysis
PPG	Photoplethysmography
RMSE	Root-mean-square error
ROI	Region of interest
RR	Respiratory rate
SSE	Sum of the squared errors
TI	Thermal image
